# The complete mitochondrial genome of the Critically Endangered Saba Green Iguana, *Iguana iguana* (Squamata: Iguanidae)

**DOI:** 10.1080/23802359.2023.2195510

**Published:** 2023-04-04

**Authors:** Matthijs P. van den Burg, Ana Ramón-Laca, Albert Carné Constans, Adolphe O. Debrot, David R. Vieites

**Affiliations:** a Departamento de Biogeografía y Cambio Global, Museo Nacional de Ciencias Naturales, Madrid, Spain; b Burg Biologica, Den Haag, The Netherlands; c Science and Business S.L., Edificio Twin Golf A bajo 2, Las Rozas, Spain; d BioCoRe S. Coop. C/Villamanín 50 Local, Madrid, Spain; e Wageningen Marine Research, Wageningen Research, Den Helder, The Netherlands

**Keywords:** Caribbean, mitogenome, next generation sequencing, Oxford Nanopore Technology, phylogeny

## Abstract

The populations of native iguanas in the Caribbean Lesser Antilles are threatened by the wide occurrence and spread of non-native iguanas. Until recently, competitive hybridization was not believed to threaten the Saba Green Iguana, a subpopulation of *Iguana iguana* (Linnaeus, 1758) from the island of Saba. However, the arrival of non-native iguanas has put the native population at risk, leading to a change in the conservation status of the Saba Green Iguana to Critically Endangered, according to guidelines from the International Union for the Conservation of Nature. Here, we generated the complete mitogenome of the Saba Green Iguana using Oxford Nanopore long-read technology. The mitogenome is 16,626 bp long and has 13 protein-coding genes, 22 tRNA genes, 2 rRNA genes, and a control region (1194 bp). Noteworthy, this is only the second published mitogenome for the *Iguana iguana* species complex, despite the known high intraspecific genetic variation.

## Main text

**Figure 1.**
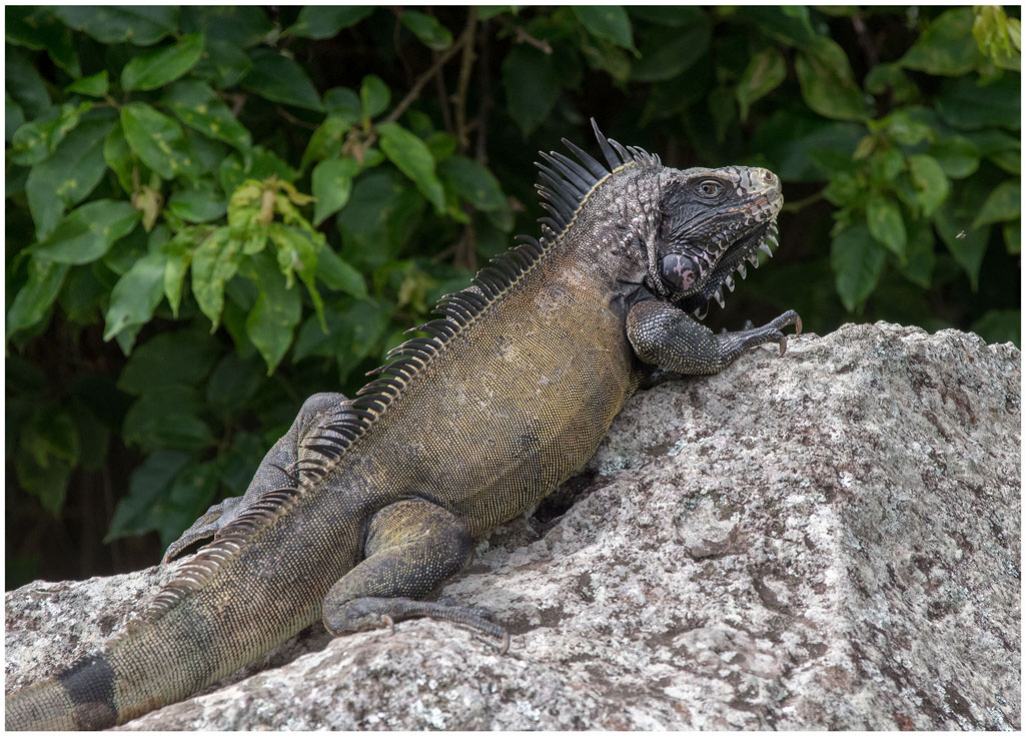
An adult Saba Green Iguana on Saba, with characteristic melanistic appearance for this population, especially between the tympanum and eye on the lateral side of the head. Photograph was taken on Saba during 2021, by Matthijs P. van den Burg.

The Saba Green Iguana is a subpopulation of *Iguana iguana iguana* (Linnaeus 1758), found only on the island of Saba in the Caribbean Lesser Antilles. It has been assessed as Critically Endangered by the International Union for Conservation of Nature (van den Burg and Debrot 2022). Like most other *Iguana* populations in the region, its survival is threatened by the recent arrival of non-native iguanas (*I. iguana*) and the resulting competitive hybridization (van den Burg et al. 2023). Some researchers consider this subpopulation to belong to the recently proposed species *Iguana melanoderma* Breuil et al. 2020 (which includes Montserrat and an undefined area of north-Venezuela), but there is no consensus on its taxonomic status. Here we follow the recommendation by the Iguana Taxonomic Working Group and retain it within *Iguana iguana iguana* until more and quantitative data are generated that support the taxonomic change (Iguana Taxonomy Working Group 2022). Here, we report on the complete mitochondrial genome of the Saba Green Iguana (Figure 1).

Despite the high diversity within the *Iguana* genus, only two complete mitochondrial genomes are available on GenBank: *Iguana delicatissima* (Miller et al. 2019) and an *Iguana iguana* specimen without georeferenced data (Janke et al. 2001). Based on published and unpublished data, we could roughly estimate the geographic location of that previously analyzed *Iguana iguana* (Stephen et al. 2013; van den Burg et al. unpublished data). Reference data, including multiple samples from Brazil, Colombia, French Guyana, Suriname, and Venezuela, suggest that the Janke et al. (2001) sample originated from the eastern side of the Andes and south of the Cordillera de Mérida. The lowest uncorrected pairwise distances to georeferenced samples were found in Bojowani (Venezuela), Trinidad (Trinidad and Tobago), and Alter do Chão (Brazil).

## Materials and methods

In 2021, we collected a blood sample (17.62636°N, −63.22746°E) stored in Longmire fieldbuffer (Longmire et al. 1997) during a population assessment on Saba, Caribbean Netherlands (van den Burg et al. 2022). This sample is at the Museo Nacional de Ciencias Naturales (Madrid, Spain; https://www.mncn.csic.es/es, lab of D. R. Vieites, vieites@mncn.csic.es), under the number SAB01. There, for DNA extraction, we used a modified phenol/chloroform protocol. We incubated 200 µl of blood in Longmire at 95 °C for 5 min to lyse cells. Samples were allowed to cool to room temperature. We then followed the extraction protocol as outlined by Ramón-Laca et al. (2021). Finally, DNA was resuspended in 100 µl of TE buffer.

To amplify the complete mitochondrial genome, we used custom-developed primers by DRV, amplifying the mt genome in two overlapping fragments, in combination with classic 16SAL and 16SBH primers (Palumbi et al. 1991). TRNALEU_iguanid__DRV_F (5′-CTTGGTGCAACTCCAAGTAAA3-3′ with 16SBH; and TRNAGLU_iguanid_DRV_R (5′-TTTGTAGTTGAATAACAACGGTGG-3′) with 16SAL. PCRs were performed in 25 µL volume using 12.5 µL of LongAmp® Taq 2X Master Mix, 1 µL of each primer (10 µM). PCR conditions were as follows: an initial denaturation step at 94 °C for 30 s; 32 cycles at 94 °C for 30 s, annealing temperature 60 °C for 30 s, extension at 65 °C for 14 s; final extension of 10 min at 65 °C. PCR products were visualized by electrophoresis on a 1.5% agarose gel stained with Sybrgreen. The samples that presented PCR product in the form of a band on the gel were sequenced.

To sequence the PCR products, we used an Oxford Nanopore Technologies (ONT) MinION MK1C sequencer. We incorporated specific adapters for ONT sequencing into our primers, following ONT guidelines, so that the PCR products (amplicons) could be sequenced directly after PCR. Next, we generated a library by pooling the PCR products, which we subsequently sequenced. Following the manufacturer’s instructions, we loaded the prepared library into an ONT R9.4.1 flowcell for MinION. The ONT MinKNOW™ software translates the resulting electrical signals into nitrogenous bases (basecalling) in real-time, which are saved in .fast5 format, and then basecalled into a fastq file. To improve the quality of the fastq file, we used the high-precision basecalling settings of the Guppy data processing toolkit integrated into MinKNOW™. The samples were run for two hours. Retrieved reads were hereafter mapped to the *Iguana iguana* mitogenome (GenBank accession NC_002793), and visually assessed and curated within Geneious Prime (2021.1.1) (Kearse et al. 2012).

To conduct a maximum likelihood analysis, we took coding and ribosomal gene fragments from the Saba Green Iguana mtgenome, and Iguaninae mtgenomes from GenBank; NC_027089, NC_002793 (Janke et al. 2001), NC_005960 (Kumazawa 2004), EU747728.2 (Castoe et al. 2008), NC_012827, NC_012829, NC_012831, NC_012834, NC_012836 and NC_012839 (Okajima and Kumazawa 2009), NC_027261 (Leache et al. 2015), NC_026308 (Bernardo et al. 2016), KT277936 and KT277937 (MacLeod et al. 2016), MK923980 (Miller et al. 2019). Briefly, we pruned coding mtDNA gene fragments, and separately aligned all mtDNA fragments using MAFFT v.7 with default parameters (Katoh and Toh 2008). We used PartitionFinder 2.1.1 (Lanfear et al. 2016) to infer the best-fit partitioning scheme and models, using the following parameters: linked branch length, BEAST models, AIC model selection, and a greedy schemes search algorithm. We performed RAxML maximum likelihood analyses with 10 tree searches, using the GTR + G model and the partitions obtained in PartitionFinder. We assessed the reliability of the ML tree using bootstrap analysis (Felsenstein 1985) with 1,000 replicates using RAxML v.8.0 (Stamatakis 2014).

## Results

**Figure 2.**
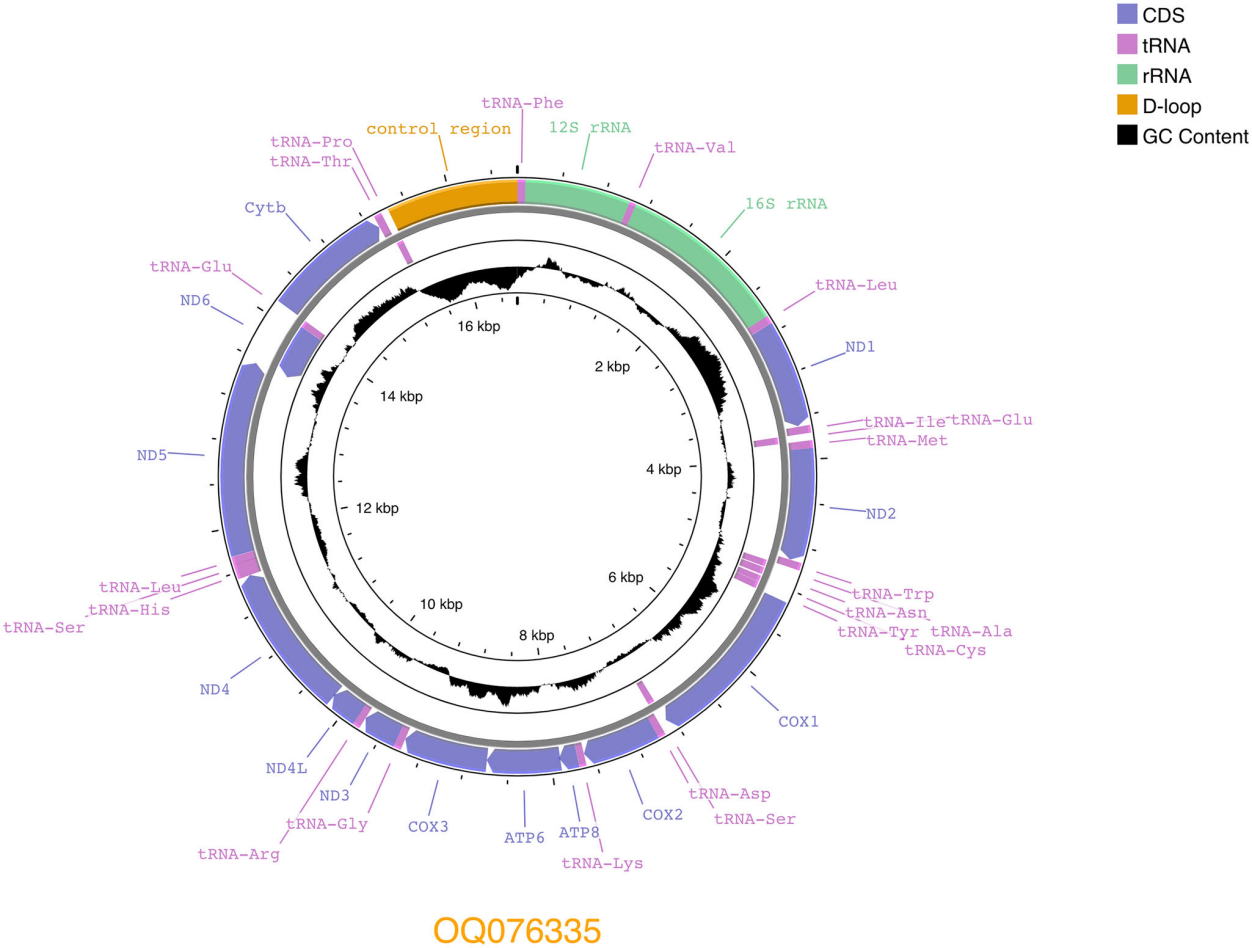
Circular view of complete annotated Saba Green Iguana (*Iguana iguana iguana*) mitogenome (GenBank accession: OQ076335), with 13 protein-coding genes, 22 tRNAs, 2 rRNAs, and a control region.

**Figure 3.**
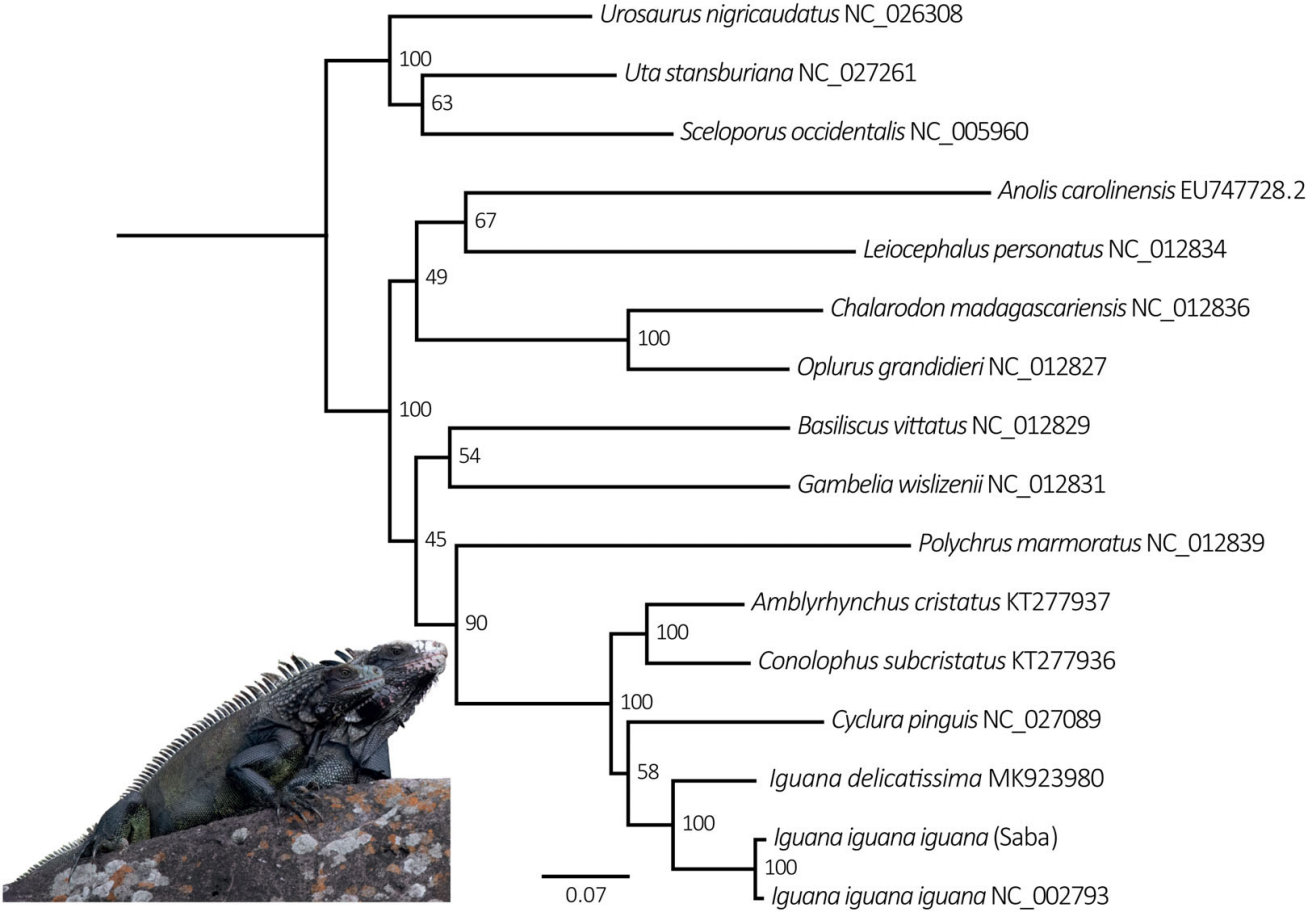
Maximum-likelihood phylogeny of aligned concatenated ribosomal and protein-coding mitochondrial loci (13.926 bp) from the Saba Green Iguana and 15 other Iguaninae taxa, performed in RAxML v. 8.0 (Stamatakis 2014) using the ML thorough bootstrap option with 1,000 replicates and 10 tree search runs. Numbers at nodes represent bootstrap support. GenBank accession numbers follow species names.

The Saba Green Iguana mitogenome (GenBank accession OQ076335) is 16,626 bp long and consists of 13 protein-coding genes, 22 tRNA and 2 rRNA, and a control region (Figure 2). It is 7 bp smaller compared to the other *I. iguana* mitogenome (NC_002793). Overall nucleotide content is, A 32.3%, C 31.7%, G 13.6% and T 22.4%. Gene arrangement is identical to that of both published *Iguana* mitogenomes (Janke et al. 2001; Miller et al. 2019). The phylogenetic analysis confirms the Saba Green Iguana’s close phylogenetic position to mainland *Iguana iguana iguana* which have *Iguana delicatissima* as sister taxa (Figure 3).

## Discussion and conclusion

Overall, uncorrected pairwise distance of the Saba Green Iguana mitogenome to the Janke et al. (2001) *Iguana iguana* mitogenome (accession NC_002793) is 1.40%, ranging between 0.84% (COX1) and 2.38% (ATP8) among protein-coding genes. The ND4 sequence of the generated mtgenome matches 100% to the only known native haplotype (“CAR2”) generated from the Saba Green Iguana population (accession HM352505: Stephen et al. 2013; Breuil et al. 2020; Mitchell et al. 2022; van den Burg et al. 2023). Continuing efforts to build a mitogenome *Iguana* reference database will aid a variety of research lines including conservation, biogeography and taxonomy, as well as the validation and range of the proposed *Iguana melanoderma* taxon through ancient DNA applications (Scheel et al. 2014).

## Supplementary Material

Supplemental Material

## Data Availability

The genome sequence data that support the findings of this study are openly available in GenBank of NCBI at [https://www.ncbi.nlm.nih.gov] under the accession no. OQ076335. The associated **BioProject**, **SRA**, and **Bio-Sample** numbers are PRJNA932829, SRX19315290, and SAMN33213536 respectively. The blood sample is stored in the lab of D. R. Vieites at the Museo Nacional de Ciencias Naturales, Madrid, Spain; code SAB01; contact corresponding author.
