# Supplementary figures and images for: The complete mitochondrial genome of the Critically Endangered Saba Green Iguana, Iguana iguana (Squamata: Iguanidae)
Source: Mitochondrial DNA B Resour. 2023 Apr 4;8(4):475–8. doi: 10.1080/23802359.2023.2195510 (PMC10075515; doi:10.1080/23802359.2023.2195510)

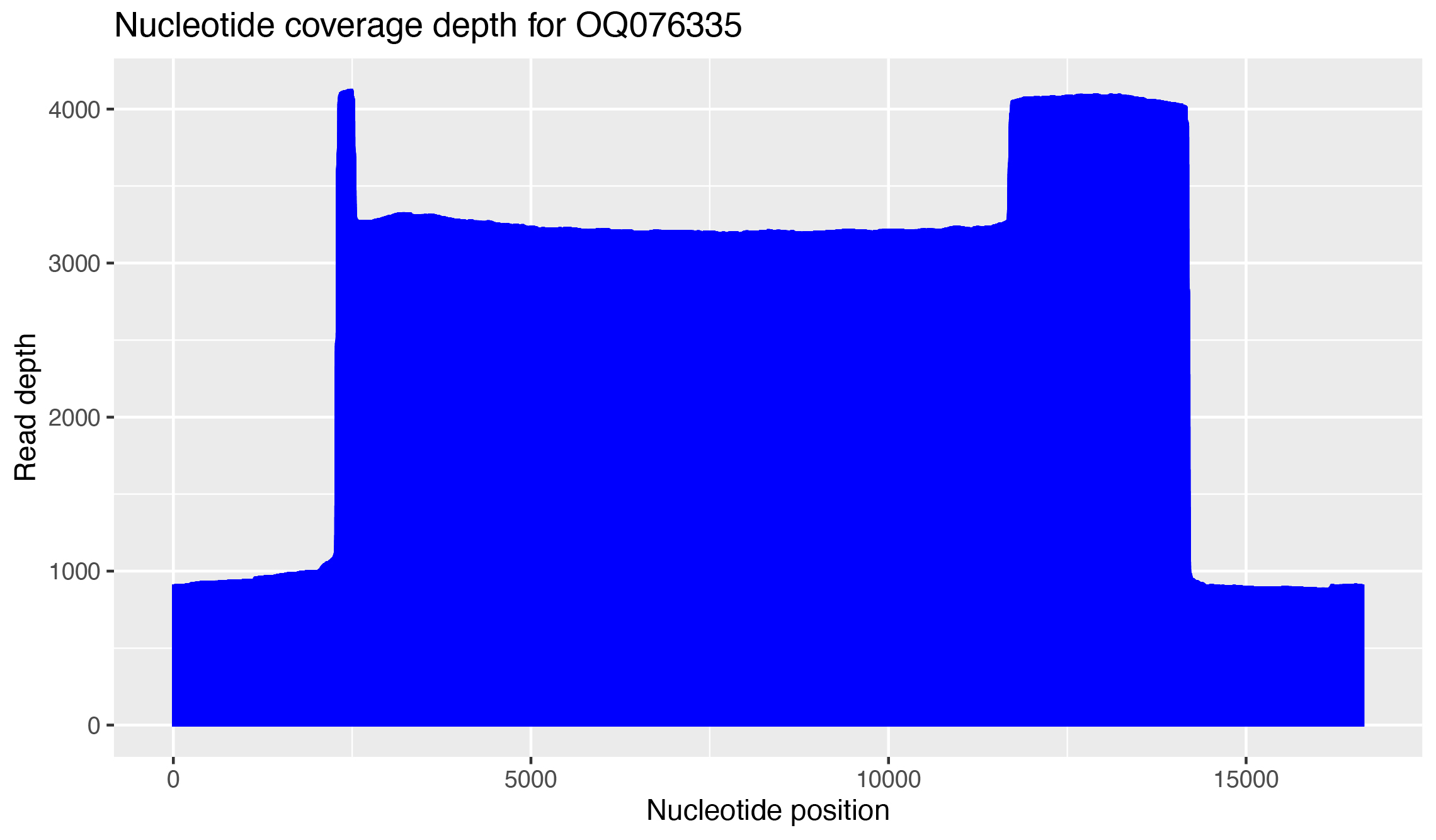

Supplement: Supplemental Material [file TMDN_A_2195510_SM9289.jpg]
